# Association of Low-Dose Quetiapine and Diabetes

**DOI:** 10.1001/jamanetworkopen.2021.3209

**Published:** 2021-05-07

**Authors:** Mikkel Højlund, Lars C. Lund, Kjeld Andersen, Christoph U. Correll, Jesper Hallas

**Affiliations:** 1 Department of Public Health, Clinical Pharmacology, Pharmacy and Environmental Medicine, University of Southern Denmark, Odense, Denmark; 2 Department of Psychiatry Aabenraa, Mental Health Services in the Region of Southern Denmark, Aabenraa, Denmark; 3 Department of Clinical Research, Psychiatry, University of Southern Denmark, Odense, Denmark; 4 Department of Psychiatry Odense, Mental Health Services in the Region of Southern Denmark, Odense, Denmark; 5 The Zucker Hillside Hospital, Department of Psychiatry, Zucker School of Medicine at Hofstra/Northwell, New York, New York; 6 Department of Child and Adolescent Psychiatry, Charité Universitätsmedizin, Berlin, Germany

## Abstract

**Importance:**

Quetiapine has been associated with increased risk of type 2 diabetes when used in medium or high doses for the treatment of severe mental disorders. It is not known whether low doses, commonly used off-label for sedative-hypnotic purposes, are also associated with increased risk of type 2 diabetes.

**Objective:**

To investigate whether there is an association between prescription of low-dose quetiapine and the risk of type 2 diabetes.

**Design, Setting, and Participants:**

This cohort study examined nationwide Danish health registers for data regarding new users of quetiapine (n = 185 938) or selective serotonin reuptake inhibitors (SSRIs) (n = 1 031 920) who were aged 18 years or older between January 1, 1998, and December 31, 2018. Individuals with schizophrenia or bipolar disorder were excluded. Quetiapine-initiators were matched 1:1 with initiators of SSRIs, using a high-dimensional propensity score (hdPS). Maximum follow-up was 5 years. Association with cumulative dose was investigated, using a case-control approach nested among quetiapine users. Data analysis was performed from May to September 2020.

**Exposures:**

Dispensing of quetiapine or SSRIs. Quetiapine prescriptions were limited to tablet strengths of 25 mg and 50 mg to focus on low-dose use.

**Main Outcomes and Measures:**

Incident type 2 diabetes was defined as first filling of an antidiabetic medication, first register diagnosis of type 2 diabetes or first hemoglobin A_1C_ measurement greater than or equal to 6.4% (≥48 mmol/mol). Incidence rates (IRs), incidence rate ratios (IRRs), and number-needed-to-harm (NNH) were calculated for full and matched cohorts using as-treated and intention-to-treat approaches. Odds ratios (ORs) were calculated for the association with cumulative quetiapine dose.

**Results:**

Altogether, 896 285 patients were included in the full cohort; 538 164 (60%) were female and the median (interquartile range) age was 47 (33-67) years. There were 57 701 low-dose quetiapine initiators and 838 584 SSRI initiators. The matched cohort consisted of 54 616 pairs. In as-treated analyses, the incidence of type 2 diabetes during treatment with low-dose quetiapine (425 cases) was 9.59 cases/1000 person-years (PY) (95% CI, 8.72-10.5/1000 PY), which was slightly higher than for SSRI users (8462 cases; IR, 8.13/1000 PY; 95% CI, 7.96-8.30/1000 PY), resulting in a significant IRR of 1.18 (95% CI, 1.07-1.30) and NNH of 684 (95% CI, 418-1873). However, the between-group difference was nonsignificant in the hdPS-matched cohort (IR, 9.49 vs IR, 9.58; IRR, 0.99; 95% CI, 0.87-1.13). The case-control analysis found no dose-response association of low-dose quetiapine with diabetes (OR for doubling of the cumulative dose: 1.02; 95% CI, 0.95-1.09; *P* = .54), but in sensitivity analyses higher daily doses were associated with diabetes (all tablet strengths: OR, 1.08; 95% CI, 1.03-1.13).

**Conclusions and Relevance:**

In this cohort study, use of low-dose quetiapine was not associated with excess risk of type 2 diabetes in comparison with SSRIs.

## Key Points

**Question:**

Is the use of quetiapine in low doses associated with increased risk of diabetes?

**Findings:**

In this nationwide cohort study that included 57 701 new users of quetiapine in low doses and without severe mental illness, the incidence of diabetes was approximately 9 cases per 1000 person-years, similar to that of a reference population treated with selective serotonin reuptake inhibitors for other psychiatric disorders.

**Meaning:**

Quetiapine used in low doses was not associated with an increased risk of diabetes among individuals with nonsevere mental illness in comparison with use of selective serotonin reuptake inhibitors.

## Teaser

This cohort study investigates whether there is an association between prescription of low-dose quetiapine and risk for type 2 diabetes among Danish adults.

## Introduction

Quetiapine is a second-generation antipsychotic medication labeled for treatment of schizophrenia, bipolar affective disorder, and as adjunctive treatment in major depression.^1,2^ Its use has increased worldwide, with quetiapine now being the most commonly prescribed antipsychotic medication among adults aged 20 to 64 years in 10 of 14 countries.^3^ In 2010, the 1-year prevalence of quetiapine use among publicly insured adults in the US was as high as 3 users per 100 inhabitants.^3^ Furthermore, several drug utilization studies have documented considerable use of quetiapine in conditions other than labeled indications, such as anxiety disorders and insomnia.^4,5,6,7^

Quetiapine is associated with a moderate risk of metabolic disturbances in comparison with other second-generation antipsychotic medications,^8,9^ and it has been linked to an increased risk of type 2 diabetes in both adolescents^10^ and adults.^11,12^ An observational study in new users of quetiapine in relatively low doses (≤200mg/d) found significant increases in fasting blood glucose with long-term treatment.^13^

Histaminergic and serotonergic antagonism plays a central role in antipsychotic-induced hyperglycemia,^9^ and quetiapine has a considerable affinity for both the H_1_- and 5-HT_2C_-receptors involved.^14^ Antipsychotic medications with high affinity of these receptors, including quetiapine, have also been associated with type 2 diabetes on the basis of adverse drug reaction reports.^15^

As quetiapine occupies H_1_- and 5-HT_2C_-receptors extensively at low doses, which are typically used for the treatment of anxiety and insomnia,^16^ we hypothesized that even low doses of quetiapine might induce metabolic disturbances leading to type 2 diabetes. An association of type 2 diabetes with low doses of quetiapine would be of particular concern given the widespread use for nonpsychotic conditions, such as insomnia. Our aim was thus to investigate the association between the prescription of low-dose quetiapine and type 2 diabetes in a controlled epidemiological design.

## Methods

### Study Design

We conducted a register-based cohort study to assess the association between prescription of quetiapine in low doses and the risk of type 2 diabetes. Access to deidentified data was approved by the Danish Health Data Authority. According to Danish legislation, no ethical approval or informed consent is needed for register-based studies. This study followed the Reporting of Studies Conducted Using Observational Routinely Collected Data for Pharmacoepidemiological Research (RECORD-PE) reporting guideline^17^ (eTable 1 in Supplement), which is an extension of the Strengthening the Reporting of Observational Studies in Epidemiology (STROBE) reporting guideline.

As mental illness, or psychological distress in general, is associated with type 2 diabetes through a multitude of mechanisms,^18,19^ we applied an active-comparator design to minimize confounding-by-indication. New users of selective serotonin reuptake inhibitors (SSRIs) were chosen as reference population, as SSRIs are frequently prescribed in nonpsychotic psychiatric conditions where low-dose quetiapine might also be used. Furthermore, SSRIs have not been associated with type 2 diabetes to the same extent as quetiapine.^11,20^

Because the effect of antipsychotics on type 2 diabetes risk may be either direct on pancreatic beta-cells, or mediated through weight gain, we analyzed the cohort in 3 ways: (1) using an as-treated (AT) approach to estimate the association with type 2 diabetes while being treated, (2) using an intention-to-treat (ITT) approach to estimate the association with type 2 diabetes among all who initiated treatment, but might stop because of other side effects (eg, sedation, lipid disturbances), while still being subject to weight gain or pancreatic dysfunction from the drug, and (3) analyzing the association of cumulative dose with type 2 diabetes, using a nested case-control approach (eTable 1 in Supplement).

### Data Sources

We collected data from 4 different Danish health care data sources with nationwide coverage. Data on prescription of quetiapine, SSRIs, and other medications were obtained from the Danish Register of Medicinal Product Statistics (DRMPS).^21^ Data on inpatient and outpatient diagnoses for outcome and comorbidity assessment were obtained from the Danish National Patient Register.^22^ Glycated hemoglobin A_1C_ (HbA_1C_) values were obtained from the Danish Laboratory Databank, which collects laboratory results from both primary care clinics and hospitals. Vital status and migration data were obtained from the Danish Civil Register.^23^ Virtually all health care in Denmark is tax-funded and freely available to all citizens, which results in near-complete coverage from these data sources.^24^ In Denmark, antipsychotic medications are only available via prescription, which means that all prescriptions from outpatient services and primary care are captured in DRMPS. Altogether, 99% of SSRI and 92% of quetiapine use is accounted for by this data source, the remainder being dispensed in hospitals.^25^

### Study Population and Exposure

We identified prescriptions of low-dose quetiapine or SSRIs in the DRMPS between January 1, 1998, and December 31, 2018, and the date of first prescription was used as the index date. We pragmatically defined low-dose quetiapine use as filling of prescriptions for 25-mg or 50-mg tablets. These tablet strengths are typically used for sedative or hypnotic purposes, and we excluded individuals who filled prescriptions for higher tablet strengths (≥100 mg) on the index date to focus on low-dose use.

Individuals who filled prescriptions for both study drugs on the index date were also excluded, together with individuals without continuous register coverage, use of other antipsychotic medications, or use of the other study drug within 365 days before the index date. Lastly, individuals with diabetes, severe mental illness, or age younger than 18 years at index date were excluded. Cohort selection is depicted in eFigure 1 in the Supplement and codes for the inclusion and exclusion criteria in eTable 1 in the Supplement.

### Outcome Definition

Incident type 2 diabetes was the defined outcome. It was defined with onset as (1) first prescription for an antidiabetic medication (Anatomical Therapeutic Chemical code [ATC]: A10), (2) first diagnosis of type 2 diabetes in registers (E10-14 in *International Statistical Classification of Diseases and Related Health Problems, Tenth Revision [ICD-10]*), or (3) first HbA_1C_ measurement of greater than or equal to 6.4% (≥48 mmol/mol).

### Statistical Analysis

#### Covariates

We used logistic regression to estimate each individual’s propensity to fill prescriptions for low-dose quetiapine. The regression model included age, sex, starting year, and the 50 most influential prescriptions or diagnoses (eTable 2 in Supplement). The latter was selected using a high-dimensional propensity score (hdPS) algorithm^26^ assessing all prescriptions and diagnoses recorded within 365 days before the index date. Hereafter, individuals were matched 1:1 using nearest-neighbor matching, allowing a caliper of 0.02 and without trimming the propensity score distribution (eFigure 2 in Supplement). For subgroup analyses, we assessed HbA_1C_ measurements within 183 days before and 7 days after the index date. Standardized mean differences (SMD) were used to assess covariate balance, with SMD less than or equal to 0.1 indicating adequate balance.^27^

#### Intention-to-Treat and As-Treated Analyses

In ITT analyses, all individuals were followed from filling of the first prescription to outcome, death, or censoring. Reasons for censoring were (1) use of higher tablet strengths of quetiapine (≥100 mg), (2) use of other antipsychotic medications, (3) use of the other study drug, (4) diagnosis of severe mental illness (eTable 1 in Supplement), (5) diagnosis of type 1 diabetes, (6) emigration, or (7) reaching 5 years of follow-up.

For as-treated (AT) analyses, follow-up was confined to the first treatment episode or censoring as described above, whichever occurred first. Treatment episodes were constructed by assigning a duration to each prescription equivalent of the number of tablets filled (assuming use of 1 tablet/d), adding a grace period of 90 days between prescriptions to account for irregular use. Gaps exceeding 90 days were considered a gap in treatment. Furthermore, we added 90 days of observation to the last prescription to capture development of type 2 diabetes occurring shortly after treatment cessation and to avoid immortal time bias.^28^

We calculated crude incidence rate ratios (IRR) and incidence rate differences (IRD) with 95% CIs for both full and hdPS-matched cohorts from the number of events per 1000 person-years of follow-up in each group. Furthermore, we calculated the number-needed-to-harm (NNH) for low-dose quetiapine-initiation as the inverse of the IRD.

#### Case-Control Analysis

To investigate the association between cumulative quetiapine dose and type 2 diabetes, we conducted a case-control analysis nested among all low-dose quetiapine users. See eMethods in the Supplement.

#### Subgroup and Sensitivity Analyses

We conducted subgroup analyses stratified on sex, age group (<65 years or ≥65 years), and presence of prediabetes at baseline (as defined in eTable 1 in the Supplement).

To test the impact of the analytical choices on the results, we conducted a number of sensitivity and supplementary analyses: (1) varying the grace period in AT analyses, (2) extending the washout window, (3) extending the maximum follow-up time, (4) excluding individuals with recurrent depression, (5) using inverse probability of censoring weights, (6) using standardized mortality ratio weights as an alternative to hdPS-matching, (8) inclusion of 100-mg quetiapine tablets, (9) inclusion of all strengths of quetiapine tablets, (10) using Z-drugs as a comparator, and (11) using olanzapine as an active assay sensitivity control exposure. For further description and rationale for these analyses, see eMethods in the Supplement.

The significance threshold was set at *P* < .05. Statistical analyses were performed using Stata/MP version 16.1 (StataCorp) from May to September 2020.

## Results

**Figure 1.**
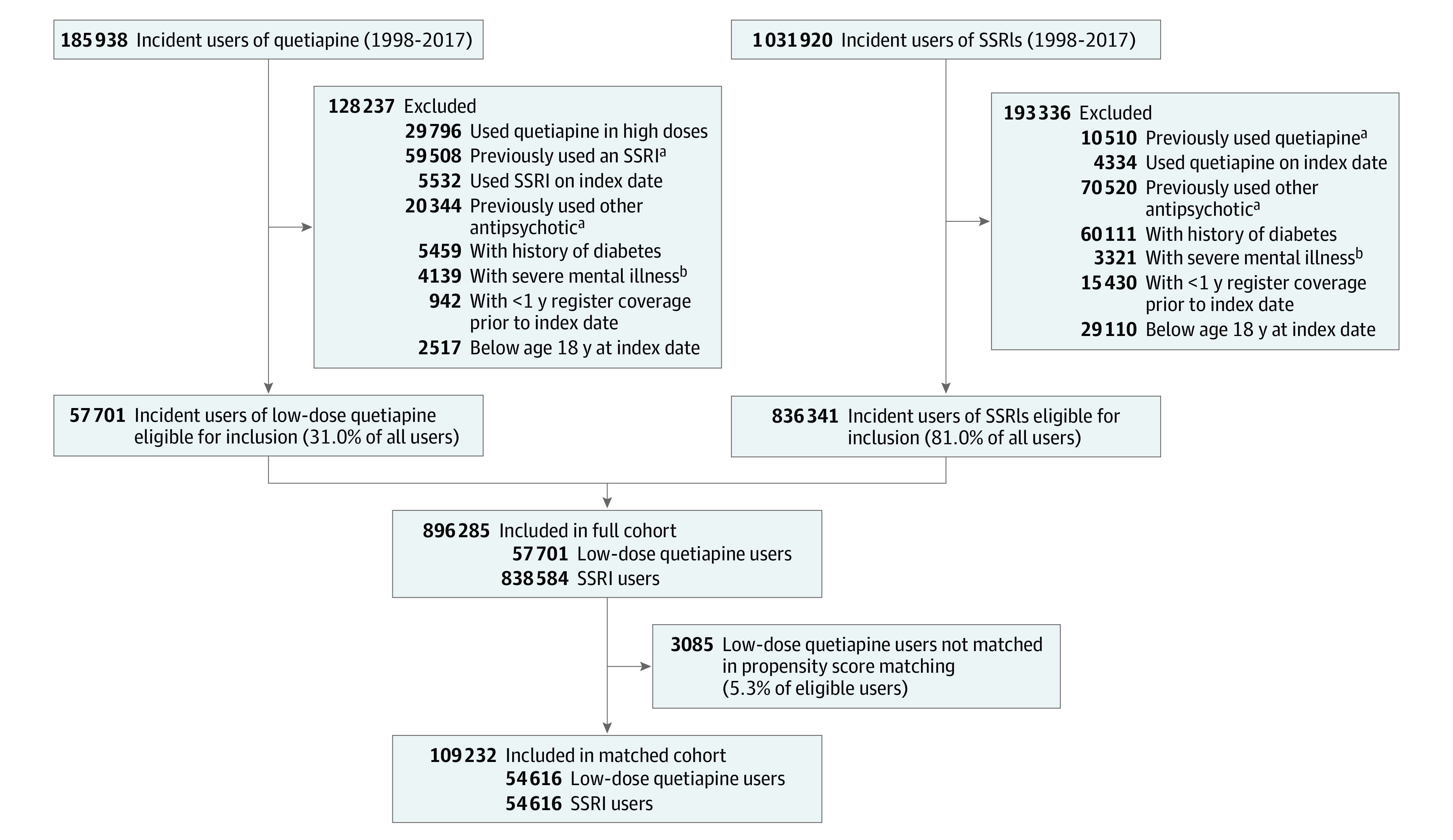
Flow Diagram of Cohort Selection SSRI indicates selective serotonin reuptake inhibitor. ^a^Within 1 year of cohort entry. ^b^Severe mental illness includes schizophrenia, schizoaffective disorder, and bipolar affective disorder.

The full cohort included 896 285 patients; 538 164 were female (60%), and the median (interquartile range [IQR]) age was 47 (33-67) years. We identified 57 701 eligible new users of low-dose quetiapine (median [IQR] age, 45 [30-64] years; 29 141 female patients [51%]) and 838 584 eligible new users of SSRIs (median [IQR] age, 47 [33-67] years; 509 023 female patients [61%]) in the DRMPS between January 1, 1998, and December 31, 2018 (Figure 1). The matched cohort consisted of 54 616 pairs with covariate balance (SMD < 0.1) on relevant characteristics, except for alcohol-related disorders and depression (Table 1). The unmatched low-dose quetiapine users were more likely to be diagnosed with depression, have alcohol-related disorders, and use mirtazapine concurrently (eTable 3 in Supplement).

**Table 1.** Baseline Characteristics of Incident Users of Low-Dose Quetiapine and Selective Serotonin Reuptake-Inhibitors in Denmark From January 1998 to December 2018

	Full cohort			hdPS-matched cohort		
	Participants, No. (%)		SMD	Participants, No. (%)		SMD
	Low-dose quetiapine	SSRI		Low-dose quetiapine	SSRI	
All	57 701	838 584		54 616	54 616	
Sex						
Female	29 141 (51)	509 023 (61)	0.21	27 383 (50)	26 237 (48)	0.04
Male	28 560 (49)	329 561 (39)		27 233 (50)	28 379 (52)	
Age, y						
Median (IQR)	45 (30-64)	47 (33-67)	<0.01	45 (29-65)	46 (29-68)	<0.01
18-64	43 349 (75)	610 368 (73)	0.05	40 898 (75)	39 357 (72)	0.06
65-79	7626 (13)	135 632 (16)	0.08	7226 (13)	8492 (16)	0.07
≥80	6726 (12)	92 584 (11)	0.02	6492 (12)	6767 (12)	0.02
Year of cohort entry						
1998-2002	83 (<1)	228 019 (27)	0.86	83 (<1)	260 (<1)	0.06
2003-2007	3622 (6)	249 081 (30)	0.64	3616 (7)	3430 (6)	0.01
2008-2012	12 820 (22)	219 460 (26)	0.09	12 622 (23)	12 358 (23)	0.01
2013-2018	41 176 (71)	142 460 (17)	1.31	38 295 (70)	38 568 (71)	0.01
Comorbidities						
Hypertension	11 835 (21)	163 686 (20)	0.02	11 095 (20)	11 981 (22)	0.04
COPD	7701 (13)	100 860 (12)	0.04	7183 (13)	6801 (12)	0.02
Heart failure	1369 (2)	22 458 (3)	0.02	1300 (2)	1482 (3)	0.02
Obesity	3504 (6)	25 383 (3)	0.15	3183 (6)	2841 (5)	0.03
Alcohol-related disorders	14 922 (26)	117 139 (14)	0.30	14 077 (26)	10 373 (19)	0.16
Major depression	12 300 (21)	47 471 (6)	0.47	10 818 (20)	5320 (10)	0.29
Recurrent depression	6225 (11)	11 905 (1)	0.40	5275 (10)	1503 (3)	0.29
Drugs used in the past year						
Digoxin	1003 (2)	22 276 (3)	0.06	967 (2)	1005 (2)	0.01
Thiazide diuretics	4292 (7)	81 780 (10)	0.08	4066 (7)	4178 (8)	0.01
Beta-blockers	6207 (11)	92 691 (11)	0.01	5802 (11)	6090 (11)	0.02
Statins	6387 (11)	75 838 (9)	0.07	5991 (11)	6237 (11)	0.01
Oral glucocorticoids	3925 (7)	58 132 (7)	0.01	3654 (7)	3795 (7)	0.01
Mirtazapine	9640 (17)	44 189 (5)	0.37	8383 (15)	8000 (15)	0.02
Antihistamines	6068 (11)	60 647 (7)	0.12	5576 (10)	4686 (9)	0.06
Hemoglobin A_1C_ at baseline						
Normal	7075 (12)	28 669 (3)	0.33	6521 (12)	8104 (15)	0.09
Prediabetes	2344 (4)	9671 (1)	0.18	2127 (4)	2552 (5)	0.04
Missing	48 282 (84)	800 244 (95)	0.39	45 968 (84)	43 960 (80)	0.10

Abbreviations: COPD, chronic obstructive pulmonary disease; hdPS, high-dimensional propensity score; IQR, interquartile range; SMD, standardized mean difference; SSRI, selective serotonin reuptake inhibitor.

Median (IQR) follow-up time in the full cohort was 1.3 (0.3-3.3) years for low-dose quetiapine users and 5.0 (2.4-5.0) years for SSRI users. For AT analyses, the median (IQR) follow-up time was 0.5 (0.3-0.8) years for low-dose quetiapine users and 0.7 (0.4-1.5) years for SSRI users. The median (IQR) number of prescriptions was 1 (1-3) for low-dose quetiapine users and 3 (1-8) for SSRI users. Among low-dose quetiapine users, 20% filled 5 or more prescriptions during their first treatment episode, and most (99%) used quantities corresponding to less than 0.25 defined daily dose (DDD) per day as calculated by the World Health Organization (eTable 4 in the Supplement). For further details on follow-up, censoring, and outcome assessment, see eTable 4, eTable 5, and eTable 6 in the Supplement.

**Figure 2.**
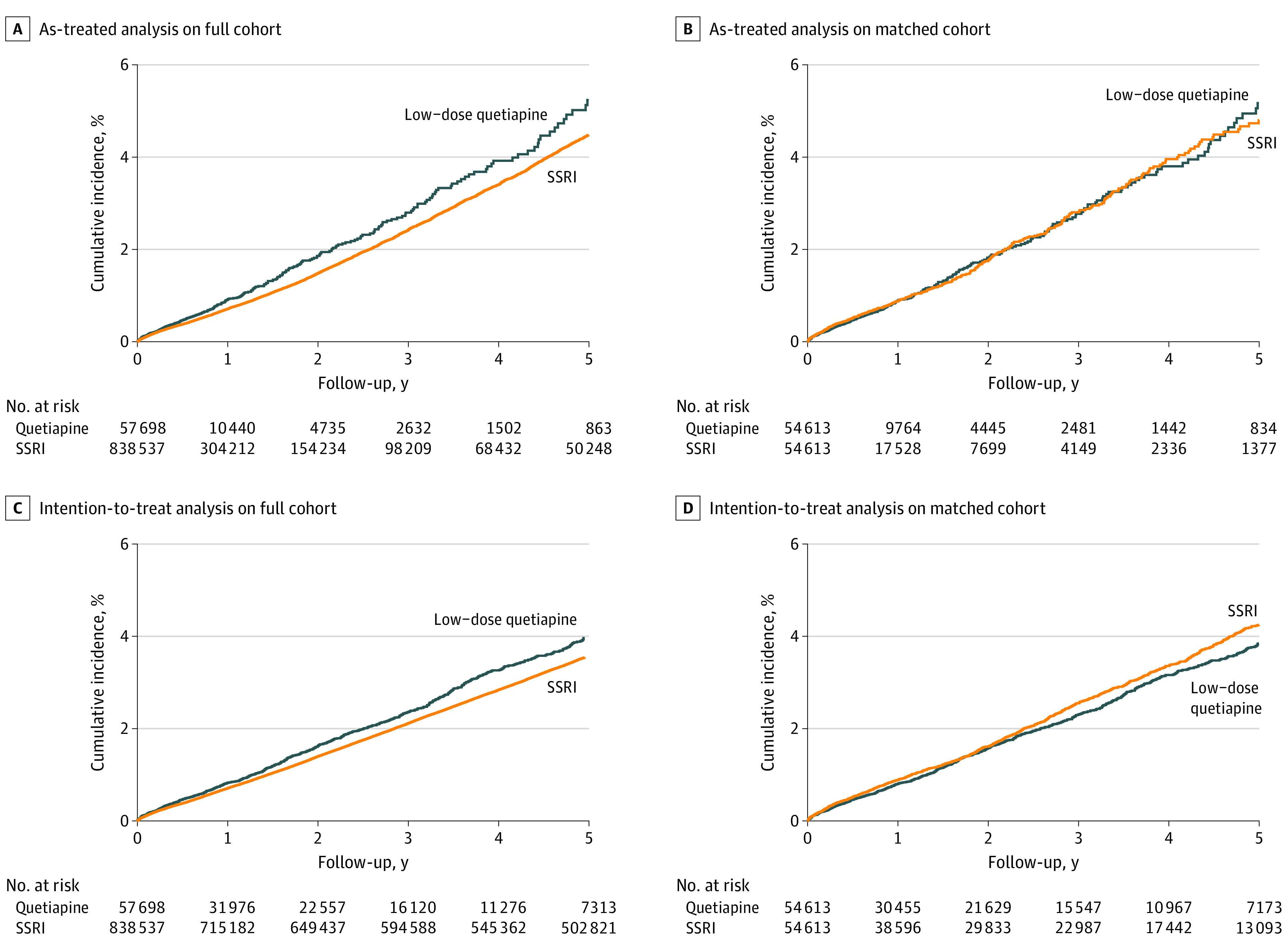
Cumulative Incidence of Diabetes After Initiation of Treatment With Low-Dose Antipsychotic or Selective Serotonin Reuptake Inhibitors (SSRIs)

Cumulative incidence of type 2 diabetes was relatively stable in both the full and matched cohorts during the follow-up period (Figure 2). Use of low-dose quetiapine was associated with a slightly elevated risk of type 2 diabetes compared with SSRIs (IRR for AT, 1.18; 95% CI, 1.07-1.30; IRR for ITT, 1.13; 95% CI, 1.06-1.21) (Table 2). However, this increased risk of type 2 diabetes was not present in the hdPS-matched cohort (IRR for AT, 0.99; 95% CI, 0.87-1.13; IRR for ITT, 0.92; 95% CI, 0.84-1.00) (Table 2).

**Table 2.** Risk of Diabetes Associated With Use of Low-Dose Quetiapine Compared With SSRIs

	Exposed, No.	Follow-up, 1000 PY	Diabetes, No.	Incidence rate, cases/1000 PY (95% CI)	Incidence rate ratio (95% CI)	Incidence rate difference (95% CI)	NNH (95% CI)
As-treated analysis							
Full cohort							
Low-dose quetiapine	57 701	44	425	9.59 (8.72 to 10.54)	1.18 (1.07 to 1.30)	1.46 (0.53 to 2.39)	684 (418 to 1873)
SSRI	838 584	1041	8462	8.13 (7.96 to 8.30)	NA	NA	NA
PS matched							
Low-dose quetiapine	54 616	42	397	9.49 (8.60 to 10.47)	0.99 (0.87 to 1.13)	−0.09 (−1.32 to 1.14)	−11537 (−760 to 876)
SSRI	54 616	58	553	9.58 (8.81 to 10.41)	NA	NA	NA
Intention-to-treat analysis							
Full cohort							
Low-dose quetiapine	57 701	110	895	8.16 (7.64 to 8.71)	1.13 (1.06 to 1.21)	0.96 (0.42 to 1.51)	1038 (664 to 2378)
SSRI	838 584	3158	22 718	7.19 (7.10 to 7.29)	NA	NA	NA
PS matched							
Low-dose quetiapine	54 616	105	837	7.97 (7.45 to 8.53)	0.92 (0.84 to 1.00)	−0.70 (−1.43 to 0.02)	−1423 (−700 to 41600)
SSRI	54 616	141	1223	8.67 (8.20 to 9.17)	NA	NA	NA

Abbreviations: NA, not applicable; NNH, number needed to harm; PS, propensity score; PY, person-years; SSRI, selective serotonin reuptake inhibitor.

In AT analysis of the full cohort, the IR of type 2 diabetes was 9.59/1000 person-years (95% CI, 8.72/1000 person-years to 10.5/1000 person-years) for those treated with low-dose quetiapine (n = 425) and 8.13/1000 person-years (95% CI, 7.96/1000 person-years to 8.30/1000 person-years) for those treated with SSRIs (n = 8462), resulting in an IRD of 1.46 (95% CI, 0.53-2.39). In the matched cohort, there were no differences in IRs for low-dose quetiapine users compared with SSRI users (IR = 9.49 vs 9.58, respectively). NNH for use of low-dose quetiapine was high in both AT and ITT analyses (NNH for AT of full cohort = 684 [95% CI, 418-1873]; NNH for ITT of full cohort = 1038 [95% CI, 664-2378]) (Table 2).

There was no clear association between cumulative dose of quetiapine (as low-dose treatment) and risk of type 2 diabetes. The OR for each doubling of the cumulative dose was 1.02 (95% CI, 0.95-1.09; *P* = .54). Furthermore, a posthoc analyses of clinically relevant dose strata found no significant increases in type 2 diabetes risk with increasing cumulative doses, and confidence intervals were overlapping for all strata (eTable 7 in Supplement).

**Figure 3.**
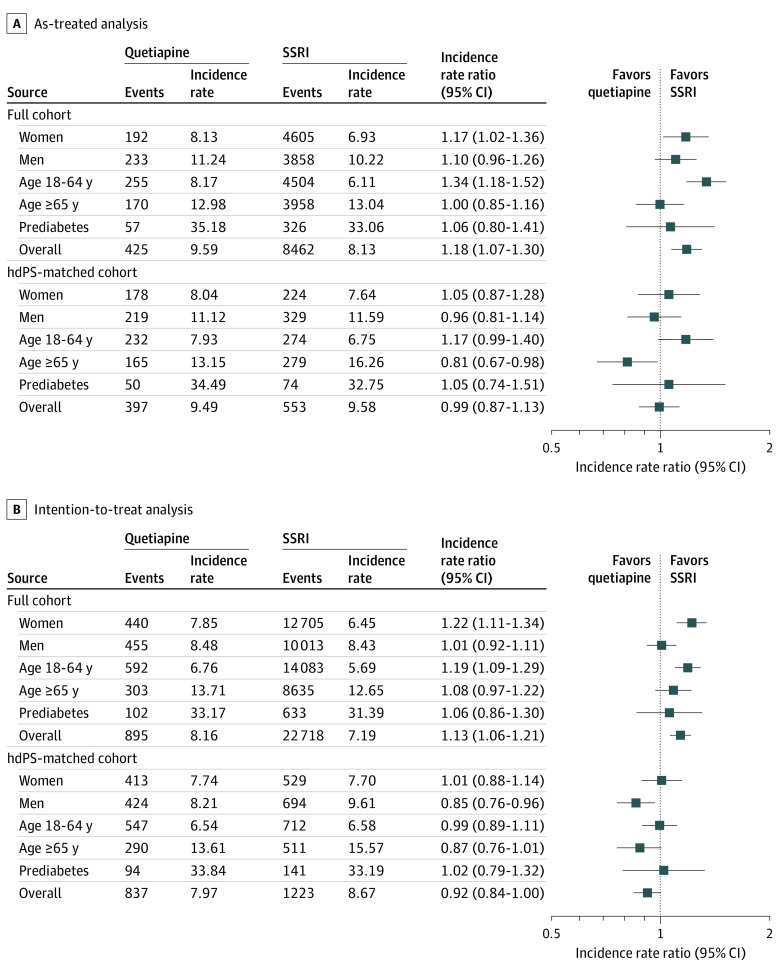
Subgroup Analysis of Association Between Diabetes and Use of Low-Dose Quetiapine or Selective Serotonin Reuptake Inhibitors (SSRIs) Prediabetes at baseline is defined as one glycated hemoglobin measurement of 5.7% to 6.3% (39-47 mmol/mol) within 6 months before and 7 days after cohort entry (only available for 9419 low-dose quetiapine users [16%] and 38 340 SSRI users [5%] in the full cohort and for 8648 low-dose quetiapine users [16%] and 10 656 SSRI users [20%] in the hdPS-matched cohort). hdPS denotes high-dimensional propensity score.

The IR of type 2 diabetes among individuals treated with low-dose quetiapine varied considerably across subgroups. In subgroup analyses of the matched cohort, female sex, age between 18 and 64 years, and prediabetes at baseline were each associated with higher IRs of type 2 diabetes than for the entire sample (Figure 3). A similar pattern was observed for SSRI users. Prediabetes at baseline was associated with the highest IRs observed for both users of low-dose quetiapine (33.8-34.5 cases/1000 person-years) and SSRIs (32.8-33.2 cases/1000 person-years).

Including higher tablet strengths in the exposure definition for quetiapine increased the association with type 2 diabetes, although most markedly when including tablets up to 400 mg (eFigure 3 in the Supplement). A similar association was also found in supplementary case-control analyses including higher tablet strengths (doubling of dose: OR, 1.08; 95% CI, 1.03-1.13) (eTable 7 in Supplement). Varying the grace period in AT analyses, extending washout period or maximum follow-up, or excluding individuals with recurrent depression was not associated with different results from the main analysis (eFigure 4 in the Supplement). Application of inverse probability of censoring weights and standardized mortality ratio weights in the main analysis did not suggest a considerable impact on the results from informative censoring or unmatched individuals, respectively (eTable 8 and eTable 9 in Supplement). Using Z-drugs as an alternative comparator did not prove useful, as individuals treated with Z-drugs were found to have an unexpectedly high rate of type 2 diabetes (eFigure 5 in Supplement). Finally, the use of olanzapine as an active control exposure was associated with assay sensitivity by detecting increased risk of diabetes among olanzapine users compared with SSRI users (eFigure 6 in the Supplement).

## Discussion

In this nationwide cohort study, we did not find an increased risk of developing type 2 diabetes with prescription of low-dose quetiapine compared with a psychiatrically ill reference population being prescribed SSRIs.

Considering all low-dose quetiapine users, we found an increased risk of type 2 diabetes associated with use of low-dose quetiapine compared with use of SSRIs (IRR = 1.18). However, this association was not present in analyses of the hdPS-matched cohort (IRR = 0.99). This difference in results likely represents an increased risk for developing type 2 diabetes among the subgroup of quetiapine users, who could not be matched to the reference population, and was unlikely attributable to the use of low-dose quetiapine itself. Individuals in this group were more likely to have a history of major depression, recurrent depression, obesity, or use of mirtazapine or antihistamines, each characteristics that are likely to increase the risk for type 2 diabetes.

Incidence rates of type 2 diabetes were higher among both low-dose quetiapine and SSRI users than in the general Danish population. Here the incidence rate was 6.1/1000 inhabitants/y among those aged 45 to 54 years in 2011^29^ in comparison with the IR of approximately 9/1000 PY observed for both low-dose quetiapine and SSRIs. There are several explanations for this increased incidence, First, the risk of developing type 2 diabetes might be higher among individuals with psychiatric morbidity, such as depression, in which both quetiapine and SSRIs are used. Second, both medications might carry a similar, increased risk of inducing type 2 diabetes. The first explanation is supported by an increased incidence of type 2 diabetes in individuals with depression.^30^ Regarding the second explanation, both SSRIs and quetiapine have been associated with development of type 2 diabetes, but the evidence for SSRIs is conflicting and the association is probably modest.^9,20,31,32^ In direct comparison with antidepressant use, quetiapine (regardless of dosage) was associated with a moderately increased risk of type 2 diabetes (HR = 1.36).^11^

Prediabetes at baseline was associated with the highest IRs observed for both users of low-dose quetiapine (33.8-34.5 cases/1000 PY) and SSRIs (32.8-33.2 cases/1000 PY). This finding must be interpreted cautiously as the number of individuals with HbA_1C_ measurements at baseline was low in both groups. Furthermore, there was no clear difference between users of low-dose quetiapine and SSRIs and the high IR more likely reflects a natural progression from prediabetes to type 2 diabetes,^33^ regardless of exposure to medications.

We found no association of increased type 2 diabetes risk with increasing cumulative dose, when exposure was confined to use of small tablets alone. However, there was a clear association between use of higher cumulative doses and risk of diabetes, when considering higher tablet strengths as proxy for higher daily doses (OR, 1.44; 95% CI, 1.13-1.84). Therefore, the daily dose is likely to be a more important risk factor than cumulative dose alone.

This study benefits from several design characteristics: The high number of individuals allowed us to perform appropriate propensity-score matching and yield results with reasonably high confidence. Furthermore, the application of an empirically driven matching procedure, using all prescriptions and hospital contacts, ensured a high degree of confounder control, which is a major issue in observational studies of diseases with multifactorial etiology, such as type 2 diabetes. The outcome definition was improved by including HbA_1C_ measurements, when available. Lastly, we conducted multiple supplementary and sensitivity analyses to test the influence of critical analytic decisions on the results and the robustness of our primary data analysis strategy.

To our knowledge, this is the first study to examine the risk of type 2 diabetes with low-dose quetiapine treatment, specifically, using a large, nationwide cohort and sophisticated data analytic methods. Using this design, we found that the risk of type 2 diabetes with use of low-dose quetiapine is not higher than among SSRI-treated controls, although it is higher than in the general population. The exclusion of a substantial type 2 diabetes risk with low-dose quetiapine is important, given the increasing number of low-dose quetiapine users worldwide.^3,34^ Many years of critical attention to the long-term use of benzodiazepines and hypnotics is a possible driver of this increase, and could have created a new public health problem, if low-dose quetiapine were associated with considerable type 2 diabetes risk. However, the high NNH (684) suggests that this risk is likely not important for the individual user or from a public health perspective, as it will not result in a substantial number of new type 2 diabetes cases. This finding does not mean that metabolic monitoring is not important with antipsychotic treatment at any dose, as some individuals will develop type 2 diabetes during treatment and as type 2 diabetes is more prevalent in the psychiatric population than in the general population. It is also important to note that our results and conclusion pertain to use of low-dose quetiapine alone and cannot be generalized, such as to higher daily doses or concomitant use with other antipsychotics or antidepressants. These populations should be the aim of future studies and continuous monitoring of metabolic risk factors, such as body mass index, blood glucose, blood pressure and lipids, should apply to all individuals treated with antipsychotics regardless of dose or indication to identify and intervene in patients with metabolic disturbances. The high proportion of new users without HbA_1C_ measurements at the treatment initiation indicates that this screening has been insufficient, as described before.^35,36,37^ Moreover, it is unclear to what degree data from this study generalize to other countries and cultures, which is why these results should be tested in other samples.

### Limitations

Some important limitations must be acknowledged. There is no obvious comparator with low-dose quetiapine. Other antipsychotic medications commonly used in low doses, such as olanzapine or risperidone, are also associated with metabolic disturbances,^38^ and not used off-label to the same extent as quetiapine.^6,34^ SSRIs are not an ideal comparator because of their association with weight gain^39^ and metabolic disturbances.^32,40^ However, these associations are likely to be inflated from population-based comparisons and not solely represent the potential obesogenic or diabetogenic effect of SSRIs.^31^ A recent study^41^ on type 2 diabetes risk in children and adolescents who initiated SSRIs compared with psychotherapy found only small increases in type 2 diabetes risk, which adds to the acceptability of SSRI as a useful and valid comparator in an adult population, as children and adolescents have a higher risk of drug-induced type 2 diabetes compared with adults.^10,42,43^ Furthermore, we tested the use of Z-drugs as an alternative comparator but found it to be unfeasible because of increased type 2 diabetes risk results. Overall, using SSRIs as a comparator allowed us to investigate the risk of quetiapine in a population with nonsevere mental illness, and to some degree separate the association with type 2 diabetes risk from psychiatric disorder/lifestyle and that of the medication. Another limitation is the low number of HbA_1C_ measurements at baseline, which limits the value of this subgroup analysis and a cautious interpretation of these results are needed. Also, information on body mass index was not available in the data sources. Inpatient or outpatient diagnoses of obesity were included in the propensity score model to take this important risk factor into account. Finally, the overall median exposure and follow-up time was still modest and longer-term observations would have further increased the confidence in our findings.

## Conclusions

The results of this cohort study suggest that there is not a significant excess risk of type 2 diabetes with use of low-dose quetiapine in comparison with SSRIs. As this study focused on low-dose quetiapine alone, future studies should focus on higher doses or concomitant use with other antipsychotics or antidepressants.

## Notes

Supplement. **eMethods.** Description of Case-control, Sensitivity, and Supplementary Analyses **eFigure 1.** Graphical Representation of Baseline Assessment and Follow-Up **eTable 1.** ICD and ATC Codes for Exposure and Outcome Variables **eTable 2.** Covariates Included in High-Dimensional Propensity Score Model **eFigure 2.** Propensity Score Distribution Before and After Matching **eTable 3.** Characteristics of Unmatched Individuals **eTable 4.** Characteristics of Follow-Up in Full and hdPS-Matched Cohort **eTable 5.** Reasons for Censoring in Full and hdPS-Matched Cohort **eTable 6.** Outcome Distribution for Full and hdPS-Matched Cohort **eFigure 3.** Supplementary Analysis Including Higher Tablet Strengths in the Exposure Definition for Quetiapine **eTable 7.** Case-Control Analysis with Different Exposure Definitions **eFigure 4.** Sensitivity Analysis Varying Grace Period, Washout Period, Maximum Follow-up, and Exclusion Criteria **eTable 8.** Supplementary Analysis Using Inverse Probability of Censoring Weights **eTable 9.** Supplementary Analysis Using Standardized Mortality Ratio-Weights **eFigure 5.** Supplementary Analysis Using Z-Drugs as Comparator **eFigure 6.** Control Analysis Using Olanzapine as Exposure
