# Supplementary material for: Association of Low-Dose Quetiapine and Diabetes
Source: JAMA Netw Open. 2021 May 7;4(5):e213209. doi: 10.1001/jamanetworkopen.2021.3209 (PMC8105749; doi:10.1001/jamanetworkopen.2021.3209)
Supplement: Supplement. — eMethods. Description of Case-control, Sensitivity, and Supplementary Analyses eFigure 1. Graphical Representation of Baseline Assessment and Follow-Up eTable 1. ICD and ATC Codes for Exposure and Outcome Variables eTable 2. Covariates Included in High-Dimensional Propensity Score Model eFigure 2. Propensity Score Distribution Before and After Matching eTable 3. Characteristics of Unmatched Individuals eTable 4. Characteristics of Follow-Up in Full and hdPS-Matched Cohort eTable 5. Reasons for Censoring in Full and hdPS-Matched Cohort eTable 6. Outcome Distribution for Full and hdPS-Matched Cohort eFigure 3. Supplementary Analysis Including Higher Tablet Strengths in the Exposure Definition for Quetiapine eTable 7. Case-Control Analysis with Different Exposure Definitions eFigure 4. Sensitivity Analysis Varying Grace Period, Washout Period, Maximum Follow-up, and Exclusion Criteria eTable 8. Supplementary Analysis Using Inverse Probability of Censoring Weights eTable 9. Supplementary Analysis Using Standardized Mortality Ratio-Weights eFigure 5. Supplementary Analysis Using Z-Drugs as Comparator eFigure 6. Control Analysis Using Olanzapine as Exposure [file jamanetwopen-e213209-s001.pdf]

Supplemental Online Content

Højlund M, Lund LC, Andersen K, Correll CU, Hallas J. Association of low-dose quetiapine and diabetes. *JAMA Netw Open.* 2021;4(5):e213209. doi:10.1001/jamanetworkopen.2021.3209

eMethods. Description of Case-control, Sensitivity, and Supplementary Analyses
eFigure 1. Graphical Representation of Baseline Assessment and Follow-Up
eTable 1. ICD and ATC Codes for Exposure and Outcome Variables
eTable 2. Covariates Included in High-Dimensional Propensity Score Model
eFigure 2. Propensity Score Distribution Before and After Matching
eTable 3. Characteristics of Unmatched Individuals
eTable 4. Characteristics of Follow-Up in Full and hdPS-Matched Cohort
eTable 5. Reasons for Censoring in Full and hdPS-Matched Cohort
eTable 6. Outcome Distribution for Full and hdPS-Matched Cohort
eFigure 3. Supplementary Analysis Including Higher Tablet Strengths in the Exposure Definition for Quetiapine
eTable 7. Case-Control Analysis with Different Exposure Definitions
eFigure 4. Sensitivity Analysis Varying Grace Period, Washout Period, Maximum Follow-up, and Exclusion Criteria
eTable 8. Supplementary Analysis Using Inverse Probability of Censoring Weights
eTable 9. Supplementary Analysis Using Standardized Mortality Ratio-Weights
eFigure 5. Supplementary Analysis Using Z-Drugs as Comparator
eFigure 6. Control Analysis Using Olanzapine as Exposure

This supplemental material has been provided by the authors to give readers additional information about their work.

© 2021 Højlund M et al. *JAMA Network Open.*

eMethods: Description of case-control, sensitivity, and supplementary analyses

Case-control analysis

The case-control analysis aimed to investigate the association between cumulative doses of quetiapine, used as low-dose treatment, and type 2 diabetes mellitus (T2DM). The case-control analysis was nested among low-dose quetiapine users and, thus, did not include a comparator group. Each T2DM case was matched on age and sex with ten controls, using risk set sampling. The observation period for each low-dose quetiapine user was similar to the follow-up period used in ITT-analyses, i.e., follow-up was confined to use of low-dose quetiapine alone and did not include time with use of SSRIs, other antipsychotics, or higher strengths of quetiapine tablets than equivalents of 25 mg or 50 mg per day.

We calculated odds ratios (ORs) for the association between cumulative quetiapine dose and T2DM, using conditional logistic regression in two ways:

1) Using cumulative quetiapine doses transformed by the binary logarithm as the independent variable. In this analysis, the OR represents the increase in risk for each doubling of the cumulative dose (pre-planned analysis).
2) Using predefined strata of cumulative dose (6.26-12.5/12.51-25/25.01-50/100.01-200/>200 DDD) as the independent variable.

Use of ≤6.25 DDD was used as reference in both approaches, as this amount is equivalent to 100 tablets of 25mg quetiapine – the smallest package marketed in Denmark.

Sensitivity analyses

To test the impact of analytical choices on the observed association between use of low-dose quetiapine and T2DM, we conducted the following six sensitivity analyses (reported in eFigures 3-4 and eTables 8-9):

1) The grace period was varied from 90 to 60 and 120 days in as-treated analyses to test the impact of grace period definition on incidence rate estimates.
2) The washout window was extended from one to five years (applying to prior use of quetiapine, SSRIs, or other antipsychotics), to assess the impact of previous use of these drugs on T2DM risk.
3) The maximum follow-up period was extended from five to 10 years, to assess if a potential long-term risk existed beyond our initial follow-up window.
4) Exclusion of individuals with history of recurrent depression from cohort entry, to assess the impact of long-term depression on T2DM risk.
5) Using inverse probability of censoring weights (IPCW) in the main analysis, to explore the magnitude of selection bias due to potential informative censoring (e.g., using higher doses quetiapine or being diagnosed with a severe mental illness). To construct ICPWs, time was partitioned into 90-day periods, and the probability of being uncensored at the beginning of each period was estimated using logistic regression analysis. The regression model included treatment status, calendar time, and all covariates from the hdPS-model (as measured at baseline). Stabilized ICPWs were then calculated as the cumulative probability of remaining uncensored, conditional on treatment status and calendar time, divided by the cumulative probability of remaining uncensored on treatment status, calendar time and baseline covariates (in the hdPS-model). We then obtained IPCW-weighted hazard ratios using pooled logistic regression analyses.
6) Using standardized mortality ratio weights (SMRW) in a Cox proportional hazards regression model as an alternative to propensity score (PS) matching. Users of low-dose quetiapine were given a weight of 1, and SSRI-users were weighted according to their PS with a weight equal to PS/(1-PS).

Supplementary analyses

To test the impact of different exposures or comparator, and to test assay sensitivity, we conducted the following four supplementary analyses:

1) Extending the exposure definition to include 100 mg quetiapine tablets. This alternative exposure definition was tested in both AT- and ITT-analyses as well as the case-control analysis of the association with cumulative dose (eFigure 3 and eTable 7).
2) Extending the exposure definition to include all strengths of quetiapine tablets. This alternative exposure definition was tested in both AT- and ITT-analyses as well as the case-control analysis of the association with cumulative dose (eFigure 3 and eTable 7).
3) Using Z-drugs as active comparator to assess the appropriateness of choosing SSRIs as the comparator in the main analyses (eFigure 5).
4) Using olanzapine as alternative exposure to test assay sensitivity of the main analyses (eFigure 6).

© 2021 Højlund M et al. *JAMA Network Open.*

eFigure 1: Graphical representation of baseline assessment and follow-up

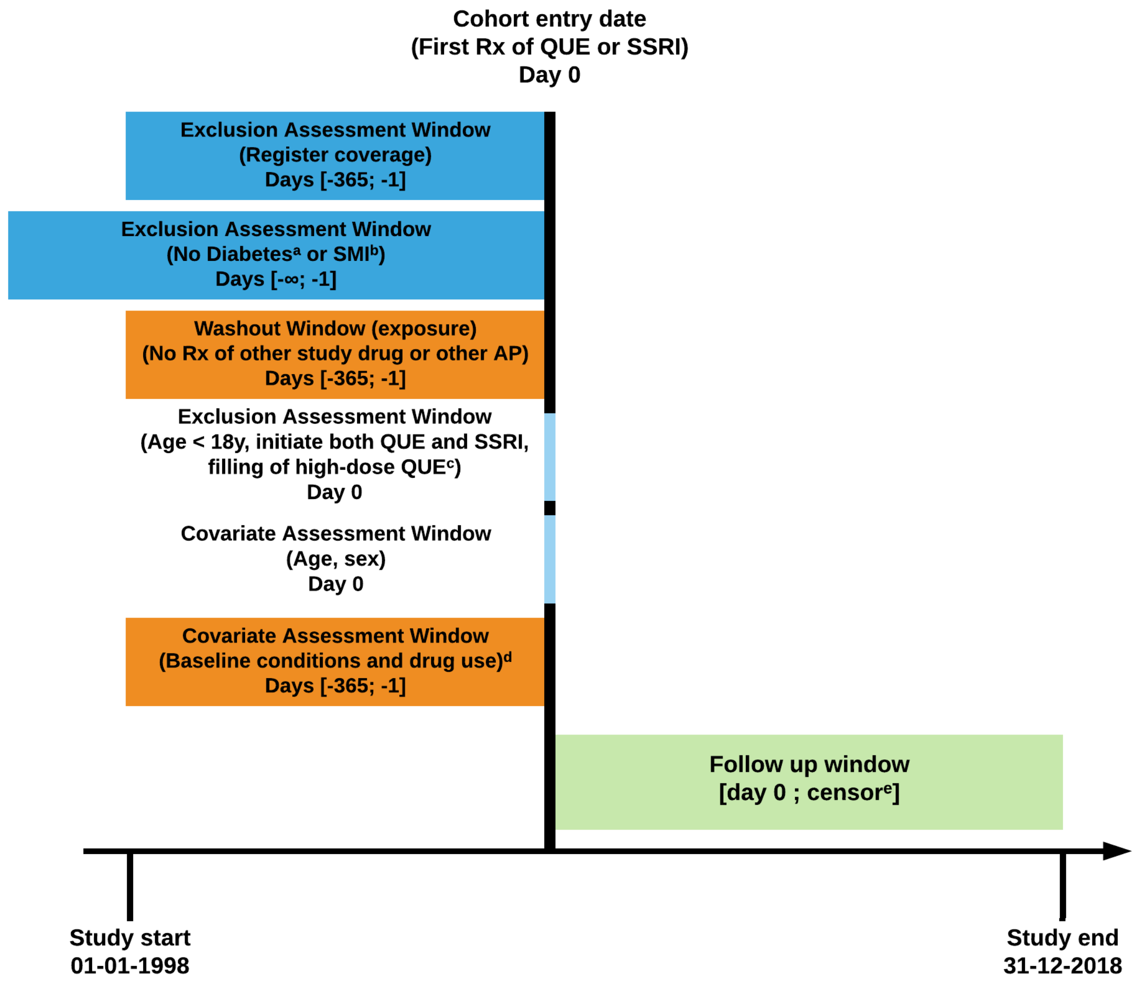

© 2021 Højlund M et al. *JAMA Network Open.*

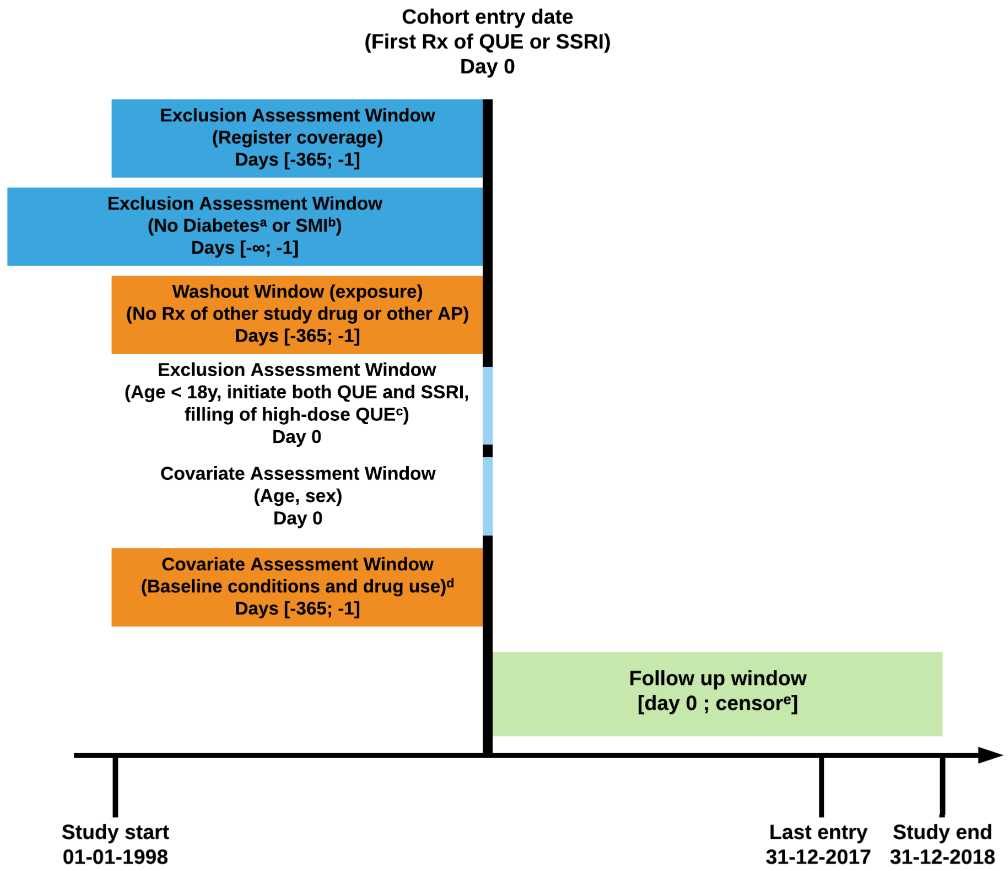

Notes: a) History of diabetes was assessed as prescriptions for antidiabetic medications, hospital diagnoses of diabetes, or blood levels of glycosylated hemoglobin above reference values, b) History of severe mental illness was defined hospital diagnoses of schizophrenia, schizoaffective disorder, or bipolar affective disorder, c) High-dose quetiapine was defined as filling of prescriptions with tablet strengths >50mg, d) Baseline covariates for the propensity score model included prescriptions and hospital diagnoses from the prior 365 days, e) Follow-up ended at the earliest of diabetes, death, emigration, end of study, five years of follow-up, or censoring for meeting baseline exclusion criteria (i.e. diagnosis of SMI, use of high-dose QUE, use of other study drug, use of other AP, or new diagnosis of type 1 diabetes mellitus).
Abbreviations: AP: Antipsychotic, Rx: Prescription, SMI: Serious Mental Illness, SSRI: Selective Serotonin Reuptake-inhibitor, QUE: Quetiapine.

© 2021 Højlund M et al. *JAMA Network Open.*

eTable 1: ICD and ATC codes for exposure and outcome variables

**Study drugs**	
Quetiapine	ATC: N05AH04
SSRIs	ATC: N06AB
**Outcome**	
Diabetes (T2DM)	ATC: A10<br>ICD-10: E10-14[a], G590, G632, G990, H280, H360, M142, N083, O240-243.<br>HbA1c ≥ 6.4% or ≥ 48mmol/mol
**Exclusion criteria**	
Severe mental illness	ICD-10: F20, 25, 30-31
Other antipsychotics	ATC: N05A (excl. N05AH04 and N05AN01)
History of diabetes	Defined as above
**Comorbidities**	
Hypertension	ICD-10: I10-13,15<br>ATC: C02, C08-09
COPD	ICD-10: J40-44<br>ATC: R03
Heart failure	ICD-10: I098-99, I110, I130, I132, I255, I420, I425-29, I43, I50
Obesity	ICD-10: E65-66
Alcohol-related disorders	ICD-10: I426, F10, K292, K70, K852, K860, T51, Y911-913, Z502, Z721<br>ATC: N07BB
Major depression	ICD-10: F32-33
Recurrent depression	ICD-10: F33
**Other medications**	
Digoxin	ATC: C01AA
Thiazide diuretics	ATC: C03A
Beta-blockers	ATC: C07
Statins	ATC: C10
Oral glucocorticoids	ATC: H02AB
Other antipsychotics	ATC: N05A (excl. N05AH04 and N05AN01)
Mirtazapine	ATC: N06AX11
Antihistamines	ATC: R06A
**Hemoglobin A1c (HbA1c)**	
Prediabetes (covariate)	HbA1c: 5.7-6.3% or 39-47 mmol/mol

a) Individuals with new diagnosis type 1 diabetes mellitus (T1DM) during follow-up were censored and were not classified as having the outcome of interest - if they had solely E10-diagnoses and prescriptions for insulin in registers, as this was considered incident T1DM. Occurrences of both E10 and other E1x-diagnoses or use of other antidiabetic medications than insulin was classified as T2DM due to the potential low validity of the T1DM-diagnosis. ATC: World Health Organization Anatomical Therapeutic Chemical classification, HbA1c: Glycosylated Hemoglobin A1c, ICD-10: World Health Organization International Classification of diseases, 10th revision.

© 2021 Højlund M et al. *JAMA Network Open.*

eTable 2: Covariates included in high-dimensional propensity score model

The 50 most common prescriptions and hospital diagnoses among the included low-dose quetiapine or SSRI-users within 12 months before cohort entry. Use of drugs or occurrence of hospital diagnoses are divided into three groups: At least one occurrence, sporadic use (i.e., more than the median number of prescriptions/hospital contacts in the cohort), and frequent use (i.e., more than the 75th percentile of prescriptions/hospital contacts in the cohort).

ATC-group or ICD-10 diagnosis	Frequency	Low-dose Quetiapine, N (%)	SSRI, N (%)	RR (cd)	RR (ce)	Multiplica-tive bias
Statins (C10AA)	One or more	5852 (10)	68270 (8)	2.37	0.73	1.025
Other special examinations and investigations (Z01, outpatient)	One or more	16242 (28)	158853 (19)	1.25	1.13	1.022
Proton pump inhibitors (A02BC)	One or more	11224 (19)	119379 (14)	1.43	1.11	1.021
Proton pump inhibitors (A02BC)	Median or more	5474 (9)	53234 (6)	1.52	1.23	1.016
Obesity (E66, outpatient)	One or more	442 (1)	3403 (0)	4.51	1.35	1.012
Other antiepileptics (N03AX)	One or more	5637 (10)	16697 (2)	1.15	3.02	1.012
Other opioids (N02AX)	One or more	8092 (14)	87408 (10)	1.33	1.42	1.012
NSAIDs (M01AE)	One or more	11797 (20)	142569 (17)	1.36	1.14	1.012
Paracetamol (N02BE)	One or more	15885 (28)	133001 (16)	1.09	1.30	1.011
Statins (C10AA)	Median or more	2278 (4)	25816 (3)	2.21	0.86	1.010
Inhaled adrenergics with corticosteroids (R03AK)	One or more	2364 (4)	23276 (3)	1.63	1.01	1.008
Non-selective monoamine reuptake inhibitors (N06AA)	One or more	3271 (6)	21024 (3)	1.26	3.28	1.008
Sleep disorders (G47, outpatient)	One or more	423 (1)	2258 (0)	2.62	1.32	1.007
Other opioids (N02AX)	Median or more	3686 (6)	34916 (4)	1.31	1.64	1.007
Medical observation (Z03, outpatient)	One or more	6459 (11)	64957 (8)	1.20	1.02	1.007
Non-selective monoamine reuptake inhibitors (N06AA)	Median or more	1959 (3)	9875 (1)	1.30	4.87	1.007
Penicillin with beta-lactamase inhibitor (J01CR)	One or more	1778 (3)	9435 (1)	1.34	1.03	1.007
Other antidepressants (N06AX)	Third quartile or more	7536 (13)	10053 (1)	1.05	13.76	1.006
Proton pump inhibitors (A02BC)	Third quartile or more	2690 (5)	28312 (3)	1.50	1.40	1.006
Other antiepileptics (N03AX)	Median or more	2887 (5)	7467 (1)	1.15	3.49	1.006
Medical observation (Z03, inpatient)	One or more	6829 (12)	64946 (8)	1.14	1.42	1.006
Drugs used in erectile dysfunction (G04BE)	One or more	1452 (3)	10703 (1)	1.45	1.20	1.006
Obesity (E66, inpatient)	One or more	325 (1)	3295 (0)	4.00	1.25	1.005
Other opioids (N02AX)	Third quartile or more	2414 (4)	21397 (3)	1.29	1.75	1.005
Beta-lactamase resistant penicillins (J01CF)	One or more	2930 (5)	29595 (4)	1.29	1.25	1.004
NSAIDs (M01AE)	Third quartile or more	2591 (4)	30257 (4)	1.52	1.27	1.004

© 2021 Højlund M et al. *JAMA Network Open.*

Other examinations and investigations (Z01, outpatient)	Third quartile or more	2307 (4)	20821 (2)	1.28	1.11	1.004
Non-selective monoamine reuptake inhibitors (N06AA)	Third quartile or more	1096 (2)	4405 (1)	1.27	6.50	1.004
Angiotensin II antagonists (C09CA)	One or more	2252 (4)	28962 (3)	1.81	0.75	1.004
ACE inhibitors and diuretics (C09BA)	One or more	917 (2)	10962 (1)	2.20	0.81	1.003

eTable 2: Covariates included in high-dimensional propensity score model (continued)

**ATC-group or ICD-10 diagnosis**	**Frequency**	**Low-dose Quetiapine, N (%)**	**SSRI, N (%)**	**RR (cd)**	**RR (ce)**	**Multiplica-tive bias**
Dihydropyridine derivatives (C08CA)	One or more	4256 (7)	58068 (7)	1.78	0.79	1.003
Persons encountering health services (Z71, outpatient)	One or more	652 (1)	5250 (1)	1.65	1.15	1.003
Paracetamol (N02BE)	Median or more	7111 (12)	61083 (7)	1.06	1.38	1.003
Other antidepressants (N06AX)	Median or more	11224 (19)	19674 (2)	1.02	9.65	1.003
Natural opium alkaloids (N02AA)	One or more	3757 (7)	32659 (4)	1.11	1.28	1.003
Drugs used in opioid dependence (N07BC)	One or more	775 (1)	2875 (0)	1.30	4.10	1.003
Other antiepileptics (N03AX)	Third quartile or more	1558 (3)	3862 (0)	1.13	3.77	1.003
Benzodiazepine related drugs (N05CF)	Third quartile or more	2849 (5)	24687 (3)	1.14	2.44	1.003
Angiotensin II antagonists and diuretics (C09DA)	One or more	1251 (2)	15834 (2)	2.03	0.77	1.003
Inhaled adrenergics with corticosteroids (R03AK)	Median or more	995 (2)	10246 (1)	1.56	0.93	1.003
Statins (C10AA)	Third quartile or more	792 (1)	9247 (1)	2.04	1.09	1.003
Natural opium alkaloids (N02AA)	Third quartile or more	1062 (2)	7728 (1)	1.30	1.71	1.003
Beta blocking agents, selective (C07AB)	Median or more	2139 (4)	28583 (3)	1.88	0.90	1.003
Benzodiazepine related drugs (N05CF)	Median or more	4747 (8)	46010 (5)	1.09	2.20	1.003
Beta blocking agents, selective (C07AB)	Third quartile or more	1182 (2)	14452 (2)	1.72	0.96	1.002
Imidazole and triazole derivatives (D01AC)	One or more	3944 (7)	49061 (6)	1.23	1.12	1.002
Hormonal contraceptives (G03AB)	One or more	223 (0)	8766 (1)	0.69	0.89	1.002
Use of tobacco (F17, inpatient)	One or more	213 (0)	1271 (0)	1.91	1.58	1.002
Muscle relaxants (M03BB)	One or more	1184 (2)	12217 (1)	1.33	1.16	1.002
Anticholinergics (R03BB)	One or more	1137 (2)	12992 (2)	1.46	0.88	1.002

ATC: Anatomical Therapeutic Chemical Classification, ICD-10: World Health Organization International Classification of diseases, 10th revision, RR(cd): Association between confounder and outcome as relative risk, RR(ce): Association between confounder and exposure as relative risk, SSRI: Selective Serotonin Reuptake-inhibitors.

© 2021 Højlund M et al. *JAMA Network Open.*

eFigure 2: Propensity score distribution before and after matching

A: Before propensity score matching, B: After propensity score matching (without trimming).

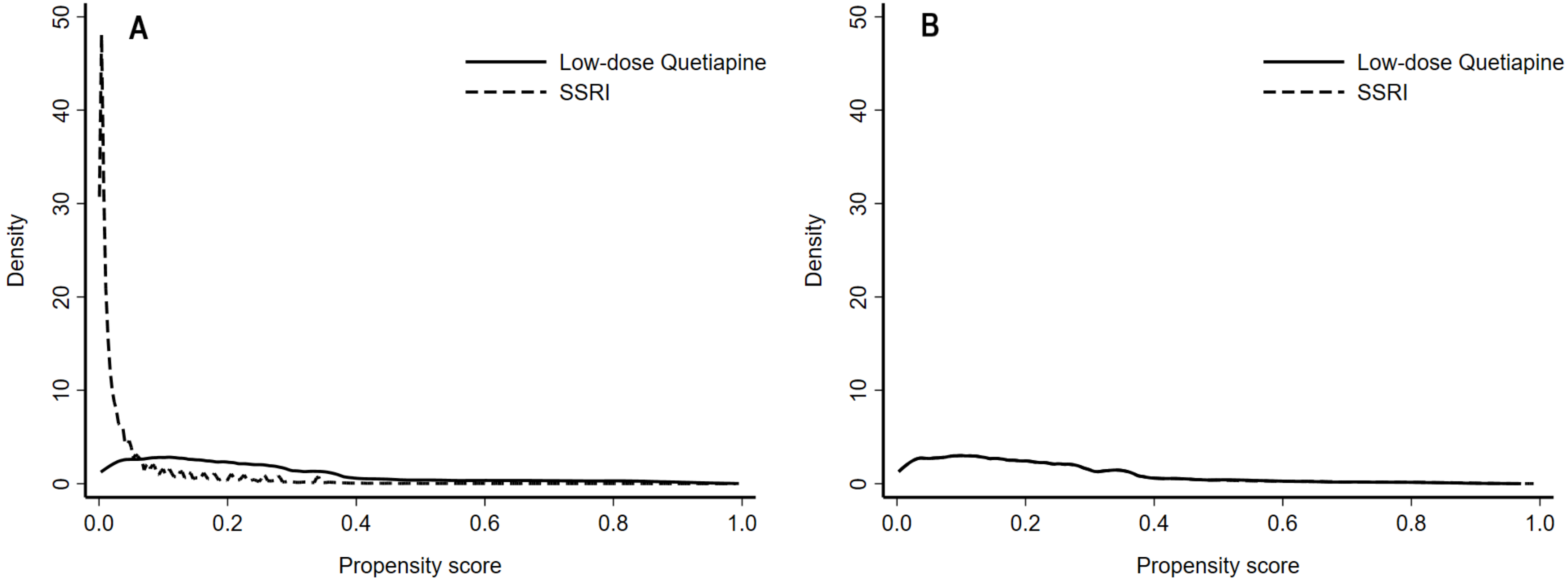

© 2021 Højlund M et al. *JAMA Network Open.*

eTable 3: Characteristics of unmatched individuals

	Individuals not included in the hdPS-matched cohort		
	**Low-dose Quetiapine**	**SSRI**	**SMD**
**All**	3,085	783,968	.
**Sex, N (%)**			.
Female	1,758 (57.0)	482,786 (61.6)	0.09
**Age, N (%)**			.
Median (IQR)	47 (36-60)	47 (33-67)	<0.01
18-64 years	2,451 (79.4)	571,011 (72.8)	0.16
65-79 years	400 (13.0)	127,140 (16.2)	0.09
80+ years	234 (7.6)	85,817 (10.9)	0.12
**Year of cohort entry, N (%)**			.
1998-2002	0	227,759 (29.1)	0.90
2003-2007	6 (0.2)	245,651 (31.3)	0.95
2008-2012	198 (6.4)	207,102 (26.4)	0.56
2013-2017	2,135 (69.2)	89,531 (11.4)	1.46
**Comorbidities, N (%)**			.
Hypertension	736 (23.9)	151,562 (19.3)	0.11
COPD	516 (16.7)	93,853 (12.0)	0.14
Heart failure	69 (2.2)	20,972 (2.7)	0.03
Obesity	307 (10.0)	21,878 (2.8)	0.30
Alcohol-related disorders	836 (27.1)	106,441 (13.6)	0.34
Major depression	1,467 (47.6)	41,996 (5.4)	1.09
Recurrent depression	941 (30.5)	10,342 (1.3)	0.87
**Drugs used in the past year, N (%)**			.
Digoxin	36 (1.2)	21,271 (2.7)	0.11
Thiazide diuretics	226 (7.3)	77,530 (9.9)	0.09
Beta-blockers	402 (13.0)	86,500 (11.0)	0.06
Statins	396 (12.8)	69,573 (8.9)	0.13
Oral glucocorticoids	270 (8.8)	54,261 (6.9)	0.07
Mirtazapine	1,251 (40.6)	36,097 (4.6)	0.95
Antihistamines	488 (15.8)	55,787 (7.1)	0.28
**Hemoglobin A1c at baseline**			.
Normal	554 (18.0)	20,565 (2.6)	0.52
Prediabetes	217 (7.0)	7,119 (0.9)	0.32
Missing	2,314 (75.0)	756,284 (96.5)	0.64

COPD: Chronic obstructive pulmonary disease, hdPS: High-dimensional propensity score, IQR: Interquartile range, SMD: Standardized mean difference, SSRI: selective serotonin reuptake inhibitor.

© 2021 Højlund M et al. *JAMA Network Open.*

eTable 4: Characteristics of follow-up in full and hdPS-matched cohort

	Full cohort		hdPS-matched cohort	
	**Low-dose Quetiapine**	**SSRI**	**Low-dose Quetiapine**	**SSRI**
	N = 57,701	N = 838,584	N = 54,616	N = 54,616
**As-treated analysis**				
Median follow-up, years (IQR)	0.5 (0.3-0.8)	0.7 (0.4-1.5)	0.5 (0.3-0.7)	0.6 (0.4-1.3)
Number of prescriptions, N (%)				
Median (IQR)	1.0 (1.0-3.0)	3.0 (1.0-8.0)	1.0 (1.0-3.0)	3.0 (1.0-6.0)
Range	1-240	1-259	1-240	1-259
1 prescription	31,147 (54)	244,649 (29)	29,791 (55)	18,570 (34)
2-4 prescriptions	15,176 (26)	249,805 (30)	14,244 (26)	17,941 (33)
5-9 prescriptions	5,308 (9)	158,321 (19)	4,937 (9)	9,682 (18)
10+ prescriptions	6,070 (11)	185,809 (22)	5,644 (10)	8,423 (15)
Average daily dose, DDD/day				
Median (IQR)	0.04 (0.03-0.07)	0.62 (0.38-0.94)	0.04 (0.03-0.07)	0.67 (0.52-1.03)
<0.25, N (%)	57,001 (99)	120,522 (14)	53,976 (99)	3,447 (6)
0.25-0.49, N (%)	671 (1)	171,168 (20)	616 (1)	9,671 (18)
0.50-0.99, N (%)	27 (0)	369,823 (44)	22 (0)	27,156 (50)
1.00+, N (%)	(n<5)	176,130 (21)	(n<5)	14,283 (26)
**Intention-to-treat analysis**				
Median follow-up, years (IQR)	1.3 (0.3-3.3)	5.0 (2.4-5.0)	1.3 (0.3-3.4)	2.3 (0.8-4.8)

Abbreviations: DDD: WHO Defined Daily Dose, hdPS: High-dimensional propensity score, IQR: Interquartile range, N: Number, SSRI: Selective serotonin reuptake-inhibitor.

© 2021 Højlund M et al. *JAMA Network Open.*

eTable 5: Reasons for censoring in full and hdPS-matched cohort

	Full cohort		hdPS-matched cohort	
	**Low-dose Quetiapine**	**SSRI**	**Low-dose Quetiapine**	**SSRI**
	N = 57,701	N = 838,584	N = 54,616	N = 54,616
**As-treated analysis**				
Death	3,422 (6)	59,603 (7)	3,302 (6)	3,511 (6)
Emigration	112 (0)	3,508 (0)	107 (0)	241 (0)
New diagnosis of T1DM	(n<5)	69 (0)	(n<5)	(n<5)
New diagnosis of SMI	752 (1)	3,119 (0)	703 (1)	233 (0)
Use of high-dose quetiapine	4,373 (8)	1,135 (0)	4,015 (7)	148 (0)
Switching to other study drug	6,540 (11)	12,625 (2)	6,322 (12)	2,261 (4)
Prescription of another AP	4,653 (8)	40,370 (5)	4,399 (8)	2,336 (4)
End of follow-up	860 (1)	50,165 (6)	831 (2)	1,371 (3)
End of study (31dec2018)	7,080 (12)	31,861 (4)	6,333 (12)	10,745 (20)
End of first treatment episode	29,484 (51)	627,736 (75)	28,207 (52)	33,217 (61)
**Intention-to-treat analysis**				
Death	4,906 (9)	98,489 (12)	4,745 (9)	5,064 (9)
Emigration	420 (1)	12,780 (2)	406 (1)	741 (1)
New diagnosis of T1DM	7 (0)	170 (0)	7 (0)	9 (0)
New diagnosis of SMI	1,082 (2)	6,536 (1)	1,013 (2)	413 (1)
Use of high-dose quetiapine	4,898 (8)	2,442 (0)	4,515 (8)	257 (0)
Switching to other study drug	9,863 (17)	24,615 (3)	9,568 (18)	3,564 (7)
Prescription of another AP	6,764 (12)	78,364 (9)	6,418 (12)	3,707 (7)
End of follow-up	7,293 (13)	502,546 (60)	7,154 (13)	13,064 (24)
End of study (31dec2018)	21,573 (37)	89,924 (11)	19,953 (37)	26,574 (49)

Abbreviations: AP: Antipsychotic drug, hdPS: High-dimensional propensity score, T1DM: Type 1 Diabetes mellitus, SMI: Severe mental illness (i.e., schizophrenia, schizoaffective disorder, and bipolar disorder), SSRI: Selective serotonin reuptake-inhibitor.

© 2021 Højlund M et al. *JAMA Network Open.*

eTable 6: Outcome distribution for full and hdPS-matched cohort

	Full cohort		hdPS-matched cohort	
	**Low-dose Quetiapine**	**SSRI**	**Low-dose Quetiapine**	**SSRI**
	N = 57,701	N = 838,584	N = 54,616	N = 54,616
As-treated analysis				
Incident T2DM	425	8,462	397	553
New diagnosis of DM	84 (20)	2,743 (32)	82 (21)	109 (20)
First prescription of antidiabetic medication	134 (32)	4,347 (51)	125 (31)	191 (35)
First measurement of elevated HbA1c	207 (49)	1,372 (16)	190 (48)	253 (46)
Intention-to-treat analysis				
Incident T2DM	895	22,718	837	1,223
New diagnosis of DM	147 (16)	6,503 (29)	144 (17)	207 (17)
First prescription of antidiabetic medication	316 (35)	12,345 (54)	296 (35)	476 (39)
First measurement of elevated HbA1c	432 (48)	3,870 (17)	397 (47)	540 (44)

Abbreviations: DM: Diabetes mellitus, HbA1c: Glycosylated hemoglobin A1c, hdPS: High-dimensional propensity score, SSRI: Selective serotonin reuptake-inhibitor, T2DM: Type 2 diabetes mellitus.

eFigure 3: Supplementary analysis including higher tablet strengths in the exposure definition for quetiapine

eFigure 3-1: Including higher tablet strengths in as-treated analyses

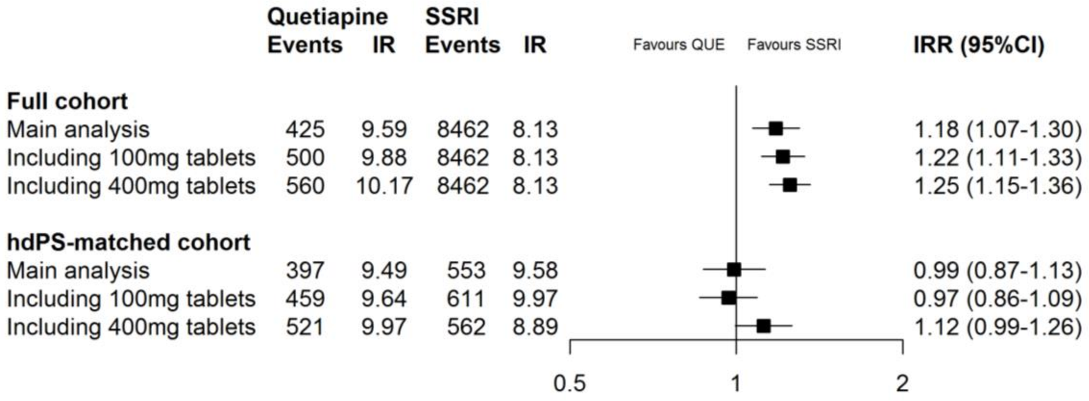

eFigure 3-2: Including higher tablet strengths in the intention-to-treat analyses

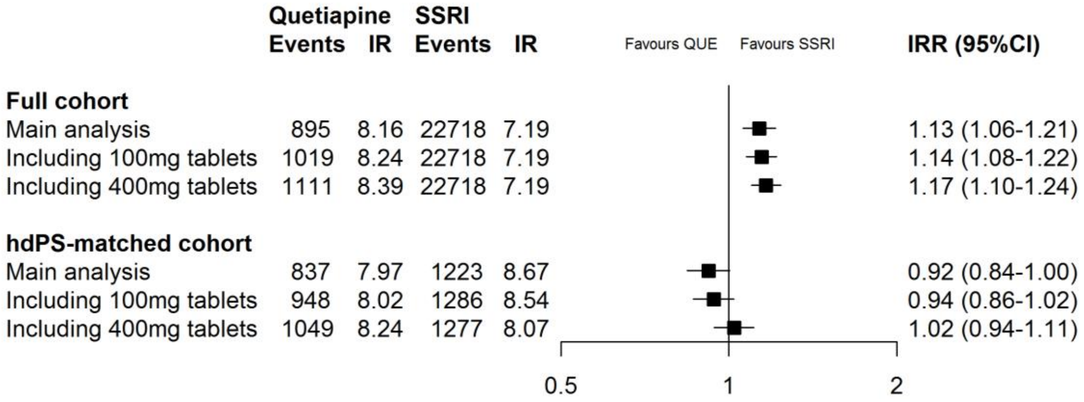

© 2021 Højlund M et al. *JAMA Network Open.*

eTable 7: Case-control analysis with different exposure definitions

	Main analysis (25-50mg tablets)			Including 25-100mg tablets[a]			Including 25-400mg tablets[a]		
**Exposure**	**Cases, No**	**Controls, No**	**Odds ratio (95%CI)**	**Cases, No**	**Controls, No**	**Odds ratio (95%CI)**	**Cases, No**	**Controls, No**	**Odds ratio (95%CI)**
Doubling of dose	895	8831	1.02 (0.95 to 1.09)	1019	10082	1.07 (1.01 to 1.12)	1111	11009	1.08 (1.03 to 1.13)
Cumulative dose, DDD	.	.		.	.		.	.	
0-6.25	381	3796	1.00 (reference)	380	3925	1.00 (reference)	380	4009	1.00 (reference)
6.26-12.5	138	1380	0.99 (0.80 to 1.23)	146	1525	0.92 (0.74 to 1.13)	150	1527	1.00 (0.81 to 1.23)
12.51-25	114	1134	1.02 (0.81 to 1.29)	127	1325	0.99 (0.79 to 1.23)	129	1391	0.97 (0.78 to 1.22)
25.01-50	109	1063	1.02 (0.80 to 1.29)	126	1224	0.99 (0.79 to 1.23)	133	1313	1.04 (0.83 to 1.30)
50.01-100	81	798	0.95 (0.73 to 1.25)	105	981	1.00 (0.79 to 1.28)	116	1168	1.07 (0.85 to 1.36)
100.01-200	46	445	1.09 (0.77 to 1.55)	73	669	1.04 (0.78 to 1.38)	90	837	1.05 (0.80 to 1.36)
>200	26	215	1.28 (0.80 to 2.05)	62	433	1.53 (1.10 to 2.11)	113	764	1.44 (1.13 to 1.84)

Notes: a) Including quetiapine tablets up to 100/400mg, but excluding individuals with severe mental disorders as in the main analysis, Abbreviations: CI: confidence interval, DDD: daily defined dose

© 2021 Højlund M et al. *JAMA Network Open.*

eFigure 4: Sensitivity analysis varying grace period, washout period, maximum follow-up, and exclusion criteria

eFigure 4-1: Varying analytical choices in as-treated analyses

	Quetiapine Events	Quetiapine IR	SSRI Events	SSRI IR	IRR (95%CI)
**Full cohort**					
Main analysis	425	9.59	8462	8.13	1.18 (1.07-1.30)
Decreasing grace period from 90 to 60 days	379	9.49	7356	8.13	1.17 (1.05-1.29)
Increasing grace period from 90 to 120 days	449	9.35	9363	8.14	1.15 (1.04-1.26)
Extending washout from 1 to 5 years	283	9.27	8256	8.11	1.14 (1.02-1.29)
Extending follow-up from 5 to 10 years	435	9.49	10035	8.51	1.11 (1.01-1.23)
Excluding recurrent depression	362	9.39	8242	8.11	1.16 (1.04-1.29)
**hdPS-matched cohort**					
Main analysis	397	9.49	553	9.58	0.99 (0.87-1.13)
Decreasing grace period from 90 to 60 days	358	9.52	486	9.48	1.00 (0.88-1.15)
Increasing grace period from 90 to 120 days	418	9.22	601	9.54	0.97 (0.85-1.09)
Extending washout from 1 to 5 years	280	9.21	406	9.71	0.95 (0.82-1.11)
Extending follow-up from 5 to 10 years	435	9.50	621	9.83	0.97 (0.86-1.09)
Excluding recurrent depression	360	9.34	502	9.42	0.99 (0.87-1.14)

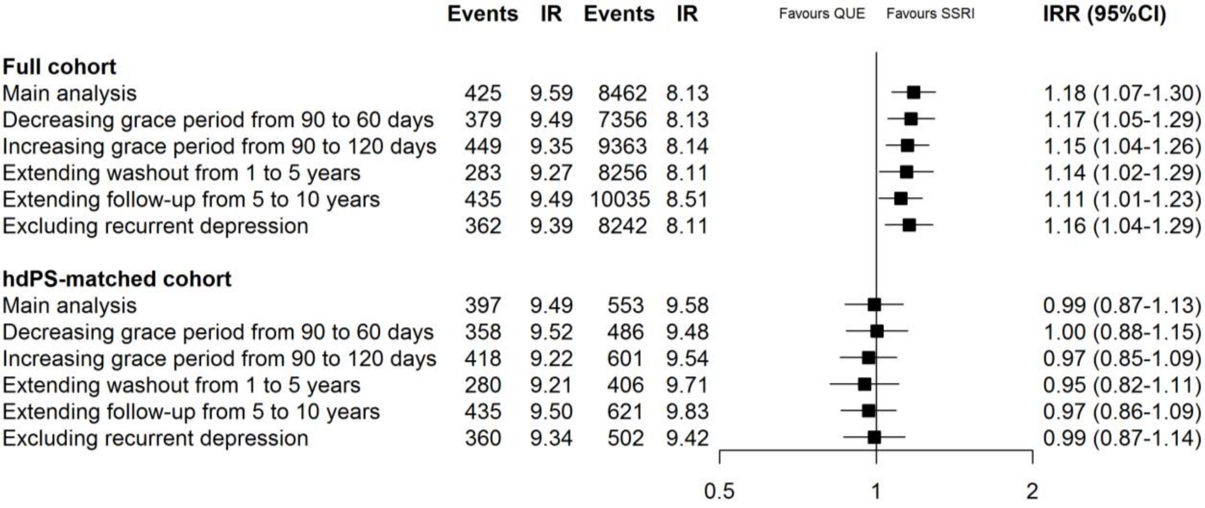

eFigure 4-2: Varying analytical choices in intention-to-treat analyses

	Quetiapine Events	Quetiapine IR	SSRI Events	SSRI IR	IRR (95%CI)
**Full cohort**					
Main analysis	895	8.16	22718	7.19	1.13 (1.06-1.21)
Extending washout from 1 to 5 years	590	7.89	22156	7.17	1.10 (1.01-1.19)
Extending follow-up from 5 to 10 years	1007	8.17	38376	7.48	1.09 (1.03-1.16)
Excluding recurrent depression	745	7.81	22083	7.18	1.09 (1.01-1.17)
**hdPS-matched cohort**					
Main analysis	837	7.97	1223	8.67	0.92 (0.84-1.00)
Extending washout from 1 to 5 years	585	7.84	838	8.34	0.94 (0.85-1.04)
Extending follow-up from 5 to 10 years	1006	8.17	1575	8.56	0.95 (0.88-1.03)
Excluding recurrent depression	743	7.79	1085	8.23	0.95 (0.86-1.04)

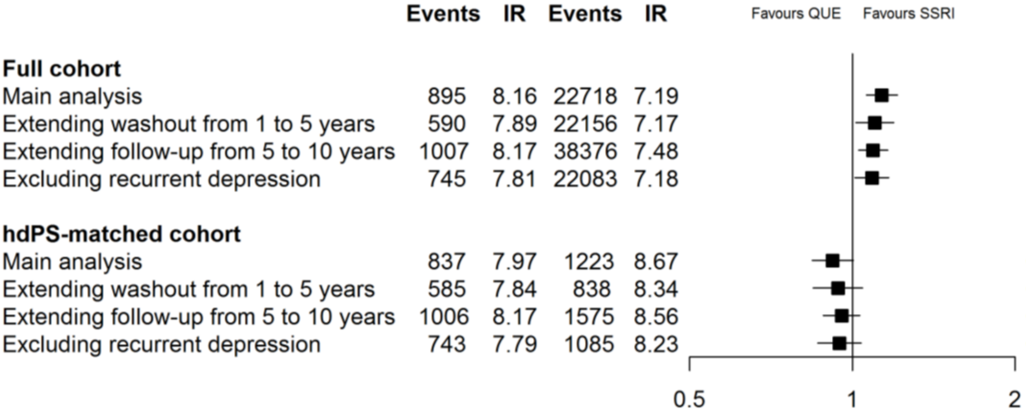

© 2021 Højlund M et al. *JAMA Network Open.*

eTable 8: Supplementary analysis using inverse probability of censoring weights

	Full cohort		hdPS-matched cohort	
	**Without IPCW**	**With IPCW**	**Without IPCW**	**With IPCW**
	HR (95%CI)	HR (95%CI)	HR (95%CI)	HR (95%CI)
As-treated analysis	1.22 (1.10-1.34)	1.18 (1.07-1.31)	1.00 (0.88-1.14)	0.98 (0.85-1.12)
Intention-to-treat analysis	1.20 (1.12-1.28)	1.27 (1.17-1.36)	0.94 (0.86-1.03)	0.92 (0.84-1.01)

Abbreviations: CI: Confidence interval, hdPS: High-dimensional propensity score, HR: Hazard ratio, IPCW: Inverse probability of censoring weights.

eTable 9: Supplementary analysis using standardized mortality ratio-weights

	Full cohort	
	**Without SMRW**	**With SMRW**
	HR (95%CI)	HR (95%CI)
As-treated analysis	1.21 (1.10-1.34)	0.98 (0.86-1.11)
Intention-to-treat analysis	1.14 (1.06-1.22)	0.94 (0.87-1.03)

Abbreviations: CI: Confidence interval, HR: Hazard ratio, SMRW: Standardized mortality ratio weights.

© 2021 Højlund M et al. *JAMA Network Open.*

eFigure 5: Supplementary analysis using Z-drugs as comparator

A: On-treatment analysis on full cohort. B: On-treatment analysis on matched cohort. C: Intention-to-treat analysis on full cohort. D: Intention-to-treat analysis on matched cohort. Exposure was benzodiazepine-related drugs (Z-drugs, ATC: N05CF, any tablet strength) and SSRIs. All other inclusion, exclusion, and censoring criteria were identical to the main analysis.

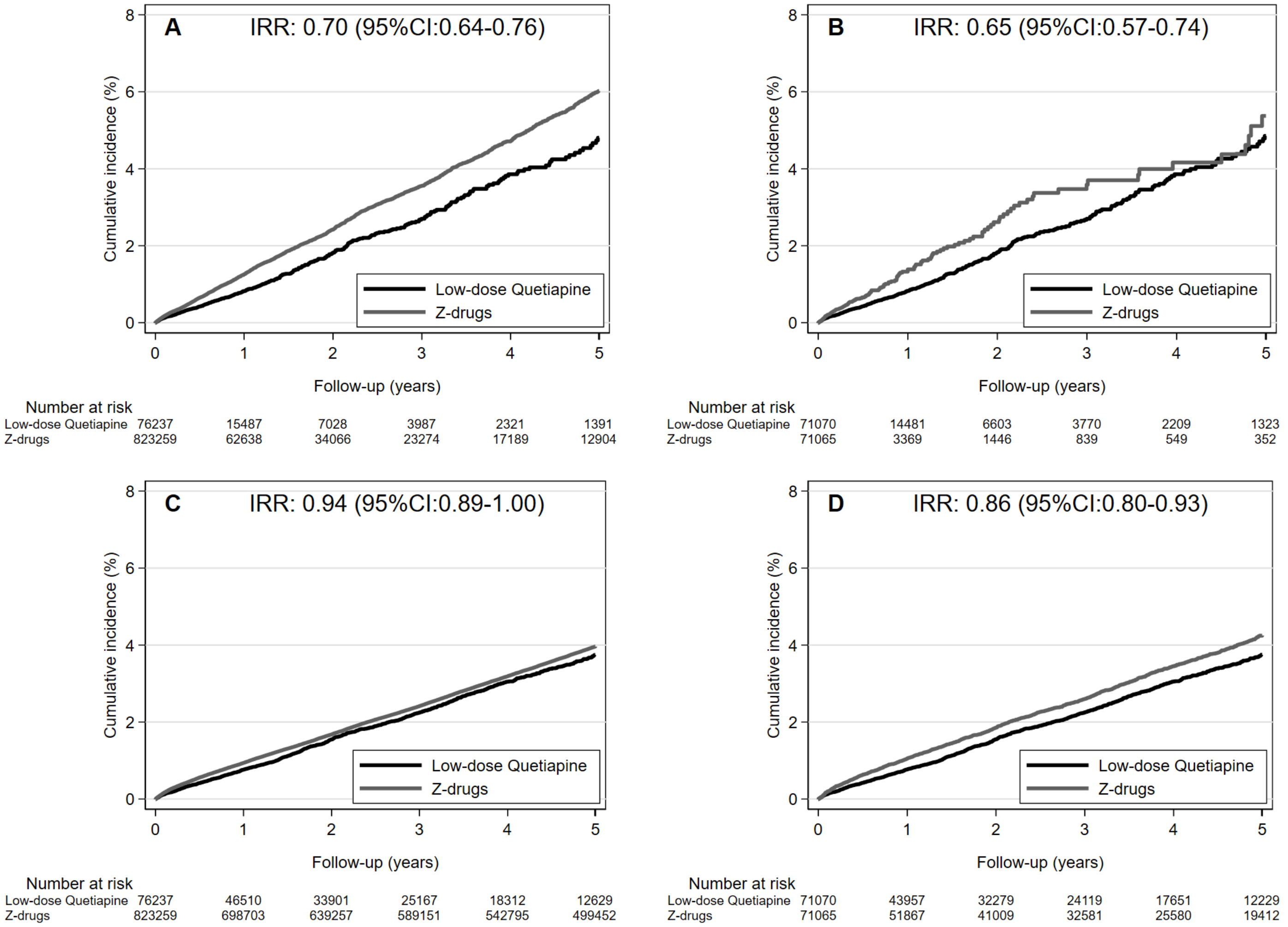

© 2021 Højlund M et al. *JAMA Network Open.*

eFigure 6: Control analysis using olanzapine as exposure

A: On-treatment analysis on full cohort. B: On-treatment analysis on matched cohort. C: Intention-to-treat analysis on full cohort. D: Intention-to-treat analysis on matched cohort. Exposure was olanzapine (N05AH03, any tablet strength) and SSRIs. All other inclusion, exclusion, and censoring criteria were identical to the main analysis.

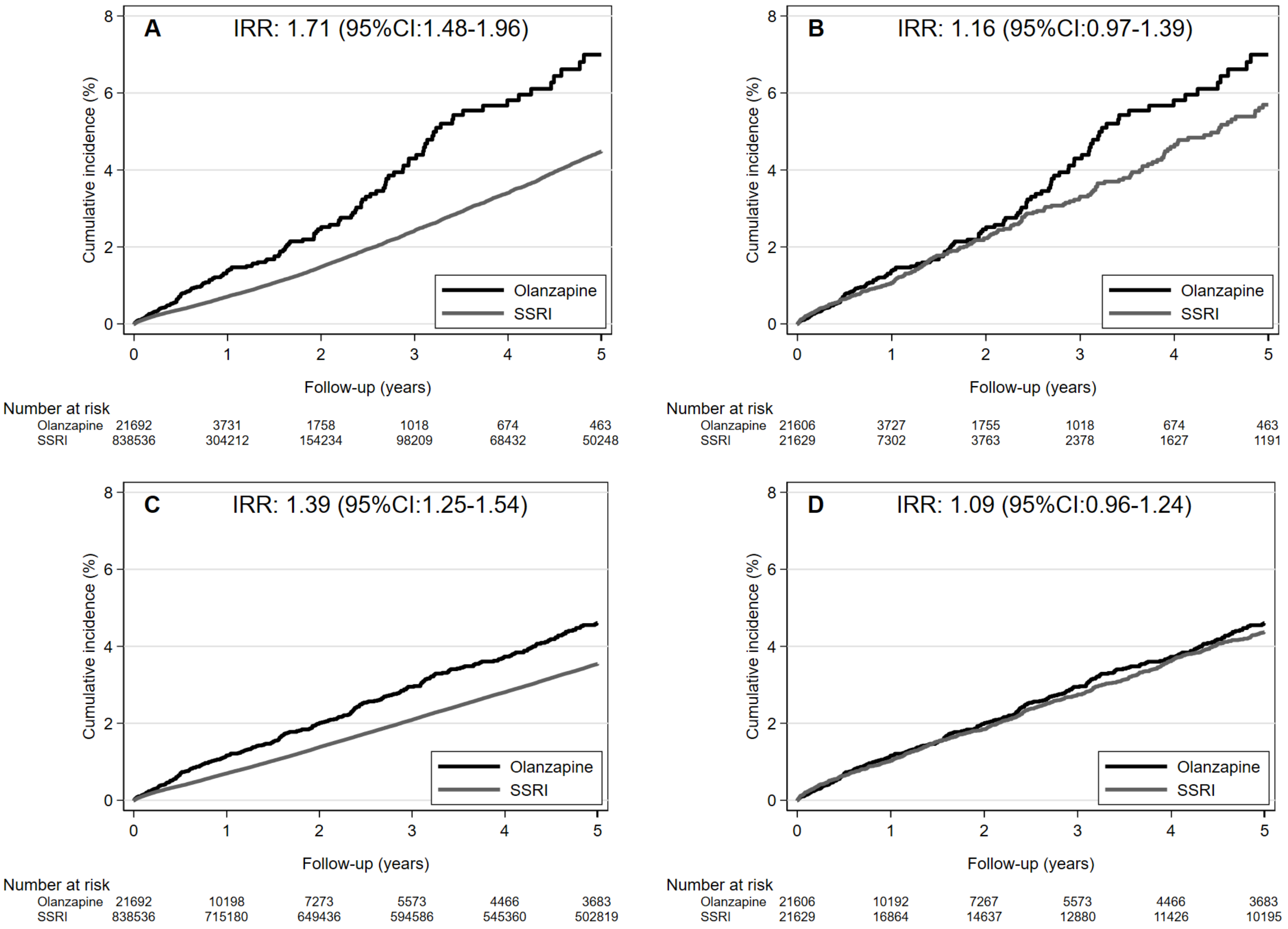

© 2021 Højlund M et al. *JAMA Network Open.*
